# Experimental preeclampsia in rats affects vascular gene expression patterns

**DOI:** 10.1038/s41598-017-14926-4

**Published:** 2017-11-01

**Authors:** Simone V. Lip, Anne Marijn van der Graaf, Marjon J. Wiegman, Sicco A. Scherjon, Mark V. Boekschoten, Torsten Plösch, Marijke M. Faas

**Affiliations:** 1 Department of Obstetrics and Gynecology, University Medical Center Groningen, University of Groningen, Groningen, The Netherlands; 2 Department of Pathology and Medical Biology, Div. of Medical Biology, University Medical Center Groningen, University of Groningen, Groningen, The Netherlands; 3 Nutrition, Metabolism and Genomics group, Wageningen University, Wageningen, The Netherlands

## Abstract

Normal pregnancy requires adaptations of the maternal vasculature. During preeclampsia these adaptations are not well established, which may be related to maternal hypertension and proteinuria. The effects of preeclampsia on the maternal vasculature are not yet fully understood. We aimed to evaluate gene expression in aortas of pregnant rats with experimental preeclampsia using a genome wide microarray. Aortas were isolated from pregnant Wistar outbred rats with low-dose LPS-induced preeclampsia (ExpPE), healthy pregnant (Pr), non-pregnant and low-dose LPS-infused non-pregnant rats. Gene expression was measured by microarray and validated by real-time quantitative PCR. Gene Set Enrichment Analysis was performed to compare the groups. Functional analysis of the aorta was done by isotonic contraction measurements while stimulating aortic rings with potassium chloride. 526 genes were differentially expressed, and positive enrichment of “potassium channels”, “striated muscle contraction”, and “neuronal system” gene sets were found in ExpPE vs. Pr. The potassium chloride-induced contractile response of ExpPE aortic rings was significantly decreased compared to this response in Pr animals. Our data suggest that potassium channels, neuronal system and (striated) muscle contraction in the aorta may play a role in the pathophysiology of experimental preeclampsia. Whether these changes are also present in preeclamptic women needs further investigation.

## Introduction

Preeclampsia is a hypertensive pregnancy disorder, which affects 2–8% of all pregnancies and is a leading cause of maternal and perinatal morbidity and mortality^1^. The development of preeclampsia is complex, but is thought to proceed in two stages. In the first stage, the placenta is poorly established (in the case of early onset preeclampsia) or poorly perfused (for late onset preeclampsia)^2^. In the second stage, proinflammatory factors, released by the “diseased” placenta into the maternal circulation, cause a systemic inflammatory response and endothelial cell activation^3^. This together leads to endothelial dysfunction, a hallmark characteristic of preeclampsia^4,5^, but possible also to an increased risk of developing heart and vascular diseases in preeclamptic women later in life^6,7^.

The endothelium plays an important role in the regulation of vascular tone by producing vasoactive factors (including: nitric oxide, endothelium-derived hyperpolarization factor [EDHF], prostacyclin, and endothelin-1)^8–10^. The endothelium-derived vasoactive factors interact with vascular smooth muscle cells to regulate vasoconstriction and relaxation. An imbalance of these vasoactive factors is associated with endothelial dysfunction^11^. During preeclampsia, an imbalance of endothelium-derived vasoactive factors occurs, with decreased nitric oxide production^12^, reduced EDHF-mediated relaxation^13^, and dysregulated prostacyclins^14^.

We recently studied endothelial function in the low dose LPS infused rat model for preeclampsia^15^. We have shown that the pregnancy-induced changes in endothelial function, such as an increased role of contractile prostaglandins and a decreased role of EDHF in acetylcholine-induced endothelial vasodilation as well as a decreased sensitivity to angiotensin II (angII), were not observed in the preeclamptic rat model^15^. Also in humans, similar changes occur in healthy pregnancy, while a lack of these changes are found in preeclamptic patients. This indicated that the model is a suitable model for studying vascular changes in preeclampsia.

Therefore, in the present study we used this low-dose LPS induced preeclampsia model and studied whole genome gene expression in the maternal vasculature, using the aorta as a model for maternal vasculature. Thus, pregnant rats were infused with a low-dose of LPS resulting in the main characteristics of preeclampsia: an increase in blood pressure, proteinuria, endothelial cell activation and an inflammatory response^16–18^. The effect of experimental preeclampsia (ExpPE) on the maternal vasculature in rats was examined by whole transcriptome expression profiling of aortic tissue using a DNA microarray and by functional contraction measurements. For control, healthy pregnant (Pr), and control non-pregnant rats (NPr) as well as low-dose LPS-infused non-pregnant rats (NPr + LPS) were used.

## Results

### Animal model

The LPS-induced preeclampsia rat model is a well-established model with the main characteristics of preeclampsia (i.e., elevated blood pressure, proteinuria, endothelial cell activation, inflammatory response)^16–18^. Maternal weight was significantly (p < 0.01) increased at day 20 of pregnancy in Pr (325.50 g ± 6.9) and ExpPE (344.22 g ± 68.5) compared to NPr (242.12 g ± 7.4) and NPr + LPS (249.75 g ± 6.9). The Pr and ExpPE rats did not significantly differ in body weight or the number of foetuses (number of foetuses: 12.1 ± 0.22 and 13.44 ± 1.0 respectively). The length of the pups was significantly (p < 0.05) smaller in ExpPE compared to Pr (31.57 mm ± 0.21 and 32.36 mm ± 0.22 respectively).

### Differences in transcriptome

Expression levels of 19,357 genes were measured in the aortas. Pr rats showed 662 significantly differently expressed genes compared to NPr control rats (p < 0.05 and a fold change >1.4 or <−1.4) (Supplementary Table S1). ExpPE showed 606 significantly differently expressed genes compared to NPr controls (Supplementary Table S2).

**Figure 1:**
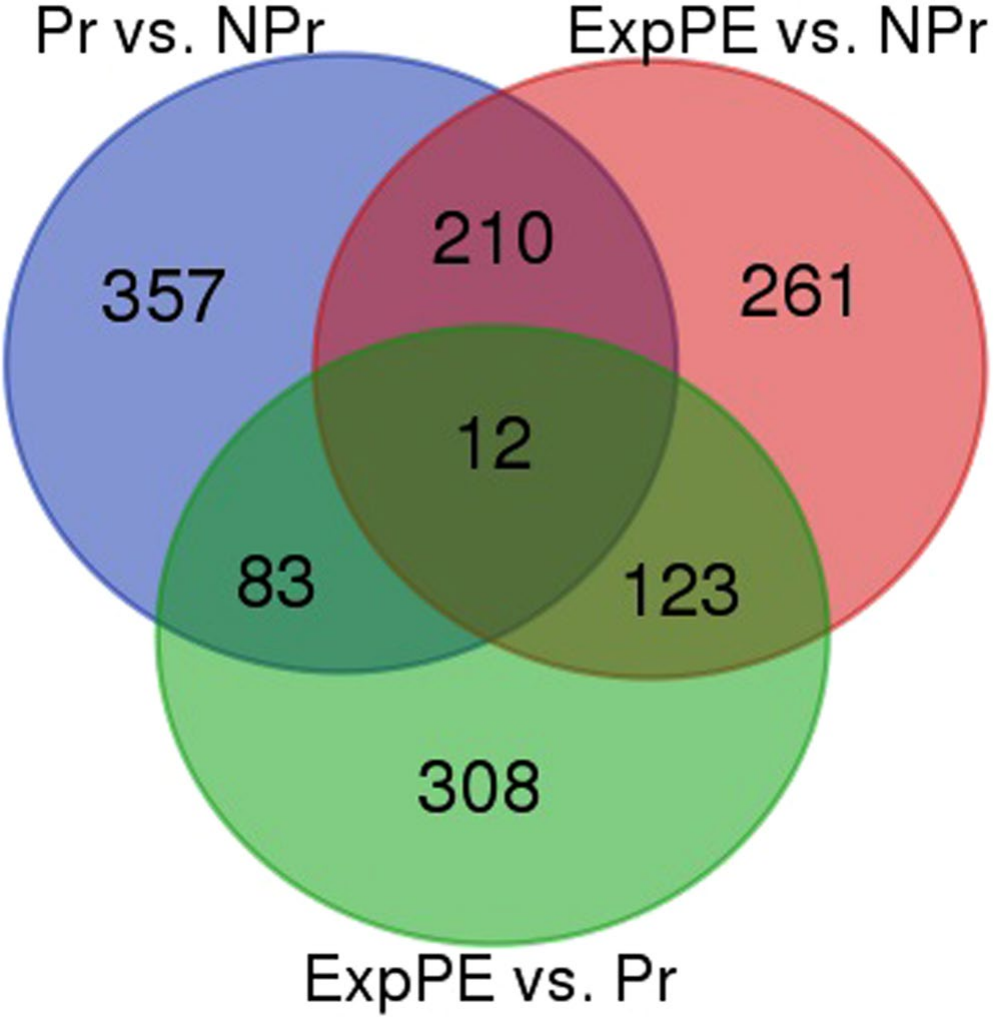
Venn diagram of aortic gene expression, representing three sets of genes which are differential expressed between the groups. The top left circle contains the differential expressed genes between healthy Pregnant (Pr; n = 5) and Non-Pregnant (NPr; n = 4) animals, the top right circle contains the differential expressed genes between Experimental Preeclamptic (ExpPE; n = 5) and NPr animals, and the bottom circle contains the differential expressed genes between ExpPE and Pr. p < 0.05, fold change <−1.4 or >1.4.

**Figure 2:**
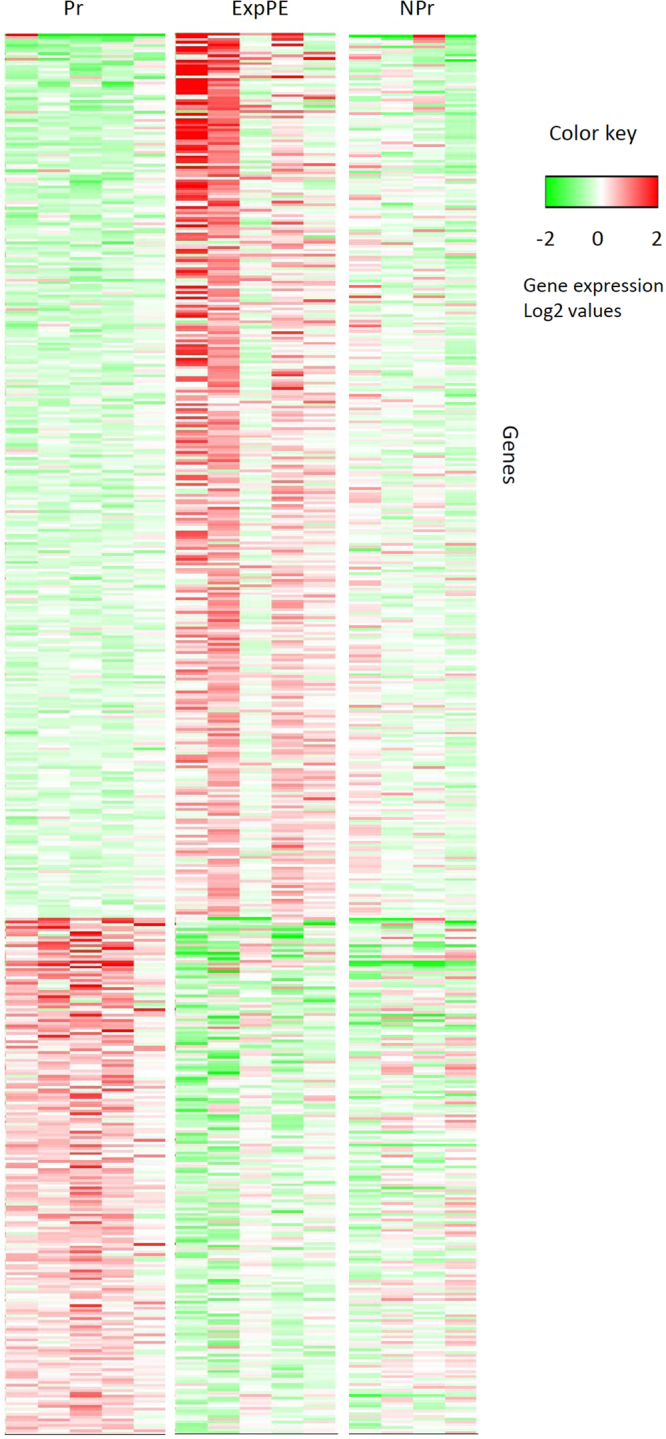
Heatmap of differentially expressed genes, ExpPE vs Pr. The average expression of all samples was used as a reference to calculate the relative gene expressions. 332 genes were significantly (p < 0.05) upregulated (fold change >1.4) and 194 genes were significantly downregulated (fold change <−1.4) in ExpPE compared to Pr. Pr = healthy Pregnant; NPr = Non-Pregnant; ExpPE = Experimental Preeclampsia.

Comparing ExpPE with healthy pregnancy revealed that 526 genes showed a significantly altered expression (Supplementary Table S3). A venn diagram shows the number of differentially expressed genes (Fig. 1). Figure 2 shows a heatmap using the 332 up- and 194 downregulated genes in ExpPE compared to Pr. The same heatmap also shows the relative gene expression values of the NPr control group. Interestingly, it appears that the upregulated genes in ExpPE are specific for ExpPE (while Pr showed gene expression levels comparable to those of NPr controls). The downregulated genes in ExpPE on the other hand, are specific for Pr (while ExpPE showed gene expression levels comparable to those of NPr controls).

### Pregnancy-induced changes in gene expression (Pr vs. NPr)

Pregnancy-induced changes were investigated by comparing gene expression levels of Pr with NPr control animals. The top 10 significantly up- and downregulated genes are listed in Table 1. The most upregulated gene is *Cxcl13* (chemokine [C-X-C motif] ligand 13), which encodes for a B-cell attracting chemokine. Number two on the list of upregulated genes is *Mmp3*, which encodes for matrix metallopeptidase 3, and is involved in tissue remodelling through the degradation of extracellular matrix^19^. The most downregulated gene in Pr is *Ucp1*, encoding for uncoupling protein 1 (mitochondrial, proton carrier).

**Table 1:** Top 10 significantly up- and downregulated genes, Pr vs. NPr.

Gene name	Fold change	p-value
*Cxcl13*	12.09	<0.001
*Mmp3*	7.61	<0.001
*Rnase1l2*	7.19	0.004
*Slfn3*	7.17	<0.001
*Irf7*	6.95	<0.001
*Mx2*	6.01	<0.001
*Oas1a*	5.62	<0.001
*Oas1b*	5.44	<0.001
*Pcsk1*	5.09	<0.001
*LOC100911190*	4.80	<0.001
*Ucp1*	−4.36	0.032
*Pnpla3*	−3.93	0.005
*Otop1*	−3.88	0.007
*Aspg*	−3.83	0.001
*Ttc25*	−3.35	0.001
*Hamp*	−3.08	0.001
*Chrnb4*	−3.05	0.048
*Fam57b*	−3.04	0.029
*Acly*	−2.98	0.011
*Gpam*	−2.94	<0.001

Gene expression was measured with a whole-genome microarray. Pr = healthy Pregnant; NPr = Non-Pregnant.

Gene Set Enrichment Analysis (GSEA) was performed to investigate gene expression changes in predefined sets of genes. The data showed that most of the significantly positively enriched gene sets, in Pr vs. NPr rats were related to the immune system, (Table 2). The top four positively enriched gene sets were “interferon signalling”, “cytokine signalling in immune system”, “interferon gamma signalling” and “interferon alpha beta signalling”.

**Table 2:** Gene Set Enrichment Analysis, Pr vs. NPr.

#	Gene sets	Normalized enrichment score	Normalized p-value	False discovery rate Q-value
1	Interferon signaling	3.15	<0.001	<0.001
2	Cytokine signaling in immune system	3.08	<0.001	<0.001
3	Interferon gamma signaling	3.05	<0.001	<0.001
4	Interferon alpha beta signaling	2.97	<0.001	<0.001

Gene expression was measured with a whole-genome microarray and analyzed by Gene Set Enrichment Analysis. Listed are the top 4 positively enriched gene sets in healthy Pregnant (Pr) compared to Non-Pregnant (NPr).

### Experimental preeclampsia-induced changes (ExpPE vs. Pr)

The top 10 significantly up- and downregulated genes in ExpPE compared to Pr are shown in Table 3. The two most highly upregulated genes in ExpPE are important in the organization of muscles: *Ttn* (Titin) and *Tnni1* (troponin I type 1 [skeletal, slow]). The most downregulated genes were *Nlrp1b* (NLR family, pyrin domain containing 1B) and *Ccl11* (chemokine [C-C motif] ligand 11). Also potentially interesting is #10 in the list, *Wnt16* (Wnt Family Member 16).

**Table 3:** Top 10 significantly up- and downregulated genes, ExpPE vs. Pr.

Gene name	Fold change	p-value
*Ttn*	8.16	0.049
*Tnni1*	3.27	0.039
*RGD1564480*	2.49	0.004
*Syngr3*	2.48	0.031
*Ugt1a1*	2.46	0.021
*Rab6b*	2.34	0.041
*Scg3*	2.30	0.025
*Snca*	2.29	0.007
*Mcpt9*	2.26	0.008
*Add2*	2.26	0.028
*Nlrp1b*	−2.97	0.023
*Ccl11*	−2.57	0.007
*LOC100359993*	−2.55	0.017
*RGD1561778*	−2.51	0.002
*LOC100361319*	−2.15	0.008
*LOC681325*	−2.12	0.005
*RT1-CE5*	−2.12	0.026
*Rpl23a*	−2.10	0.022
*Cd180*	−2.06	0.002
*Wnt16*	−2.05	0.014

Gene expression was measured with a whole-genome microarray. ExpPE = Experimental Preeclampsia; Pr = healthy Pregnant.

Below the top 10 we also found some interesting genes with regard to possible changes in vascular function, for example *Nos1* (nitric oxide synthase 1; p = 0.044, fold change = 1.67), *Edn3* (endothelin 3, p = 0.034, fold change = 1.46), and *Ang2* (angiogenin, ribonuclease A family, member 2; p = 0.010, fold change = 1.45) were upregulated in ExpPE compared to Pr and *Esm1* (endothelial cell specific molecule 1; p = 0.026, fold change −1.78) was downregulated in ExpPE compared to Pr.

**Figure 3:**
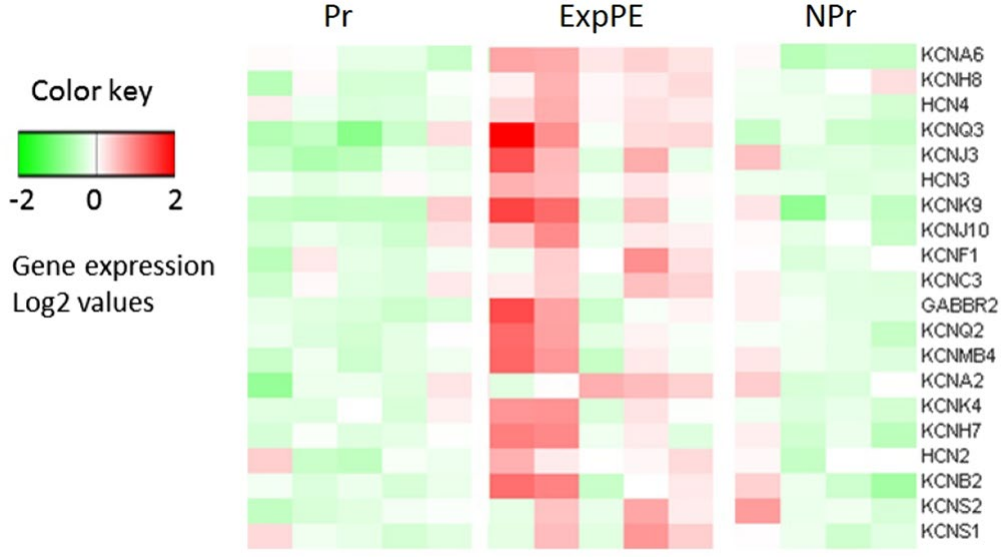
Heatmap of the 20 most contributing genes to the positive enrichment of the potassium channel gene set. The average expression of all samples was used as a reference to calculate the relative gene expressions. Pr = healthy Pregnant; NPr = Non-Pregnant; ExpPE = Experimental Preeclampsia.

GSEA was performed comparing ExpPE to Pr. The most positively enriched gene sets were “potassium channels”, “striated muscle contraction” and the “neuronal system” (Table 4). The genes that contribute the most within the potassium channels gene set are *Kcna6*, *Kcnh8* and *Hcn4*. The genes that contribute the most within the neuronal system gene set are *Kcna6*, *Cacng3* and *Syn3*, and for the striated muscle contraction gene set are *Myh8*, *Tnni1* and *Myh3*. A heatmap of the 20 most strongly contributing genes in the potassium channels gene set was generated (Fig. 3).

**Table 4:** Gene Set Enrichment Analysis, ExpPE vs. Pr.

**#**	**Gene sets**	**Normalized enrichment score**	**Normalized p-value**	**False discovery rate Q-value**
1	Potassium channels	2.40	<0.001	<0.001
2	Voltage gated potassium channels	2.40	<0.001	<0.001
3	Striated muscle contraction	2.35	<0.001	<0.001
4	Neuronal system	2.30	<0.001	<0.001

Gene expression was measured with a whole-genome microarray and analysed by Gene Set Enrichment Analysis. Listed are the top 4 positively enriched gene sets in Experimental Preeclampsia (ExpPE) compared to healthy Pregnant (Pr).

### Microarray validation

Real-time quantitative PCR (RT qPCR) was performed to validate the microarray data. Gene expression levels of 11 genes in total were evaluated. The 11 genes chosen were of most interest because they include: the top 2 upregulated genes in ExpPE vs. Pr which were also in the “striated muscle contraction” gene set. Two additional genes of the same gene set were also included, the top 5 mostly contributing genes to the enrichment of the gene set “potassium channels”, and the top 3 genes of the “neuronal system”. For 9 of the 11 genes a significant linear correlation was found between RT qPCR data and array data (Supplementary Fig. 1). For *Myh3* and *Myh8* (Supplementary Fig. 1C,D) no linear correlation was found, probably due to very low expression values which are not properly detectable by RT qPCR.

### LPS-infusion in NPr controls

**Figure 4:**
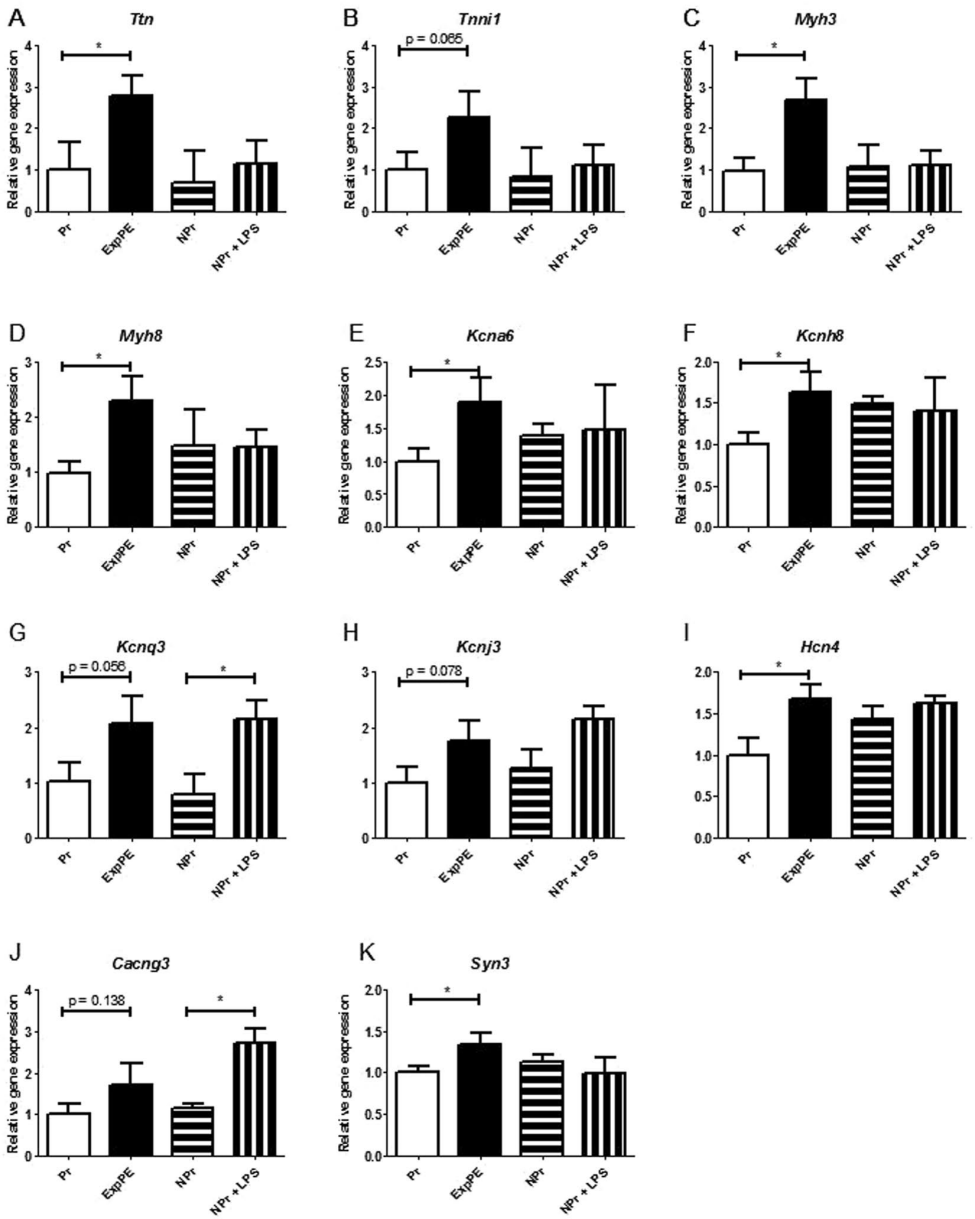
Gene expression was measured by RT qPCR of an additional control group for LPS infusion in non-pregnant animals. Expression levels of *Ttn*, *Tnni1*, *Myh3*, *Myh8*, *Kcna6*, *Kcnh8* (**A**–**F**), *Kcnj3, Hch4*, (**H**,**I**), and *Syn3* (**K**) did not differ between NPr and NPr + LPS animals. The genes *Kcnq3* (**G**), and *Cacng3* (**J**) were found differently expressed due to LPS infusion independent of pregnancy. Pr = healthy Pregnant; ExpPE = Experimental Preeclampsia; NPr = Non-Pregnant; NPr + LPS = Non-Pregnant + LPS. Data are presented as mean ± SEM. * p < 0.05.

As a control for LPS effects, expression of 11 genes (also included in the validation of the array) was measured by RT qPCR in LPS-infused non-pregnant rats (Fig. 4). *Ttn*, *Myh3*, *Myh8*, *Kcna6*, *Kcnh8* (Fig. 4A,C–F), *Hch4 (*Fig. 4I), and *Syn3* (Fig. 4K) were all significantly increased in ExpPE rats vs Pr rats, but not in NPr + LPS vs NPr. The expressions of the genes *Kcnq3* (Fig. 4G), and *Cacng3* (Fig. 4J) were significantly increased in NPr + LPS vs NPr and not in ExpPE vs Pr.

### *Ex vivo* aortic ring contractile response to KCl

**Figure 5:**
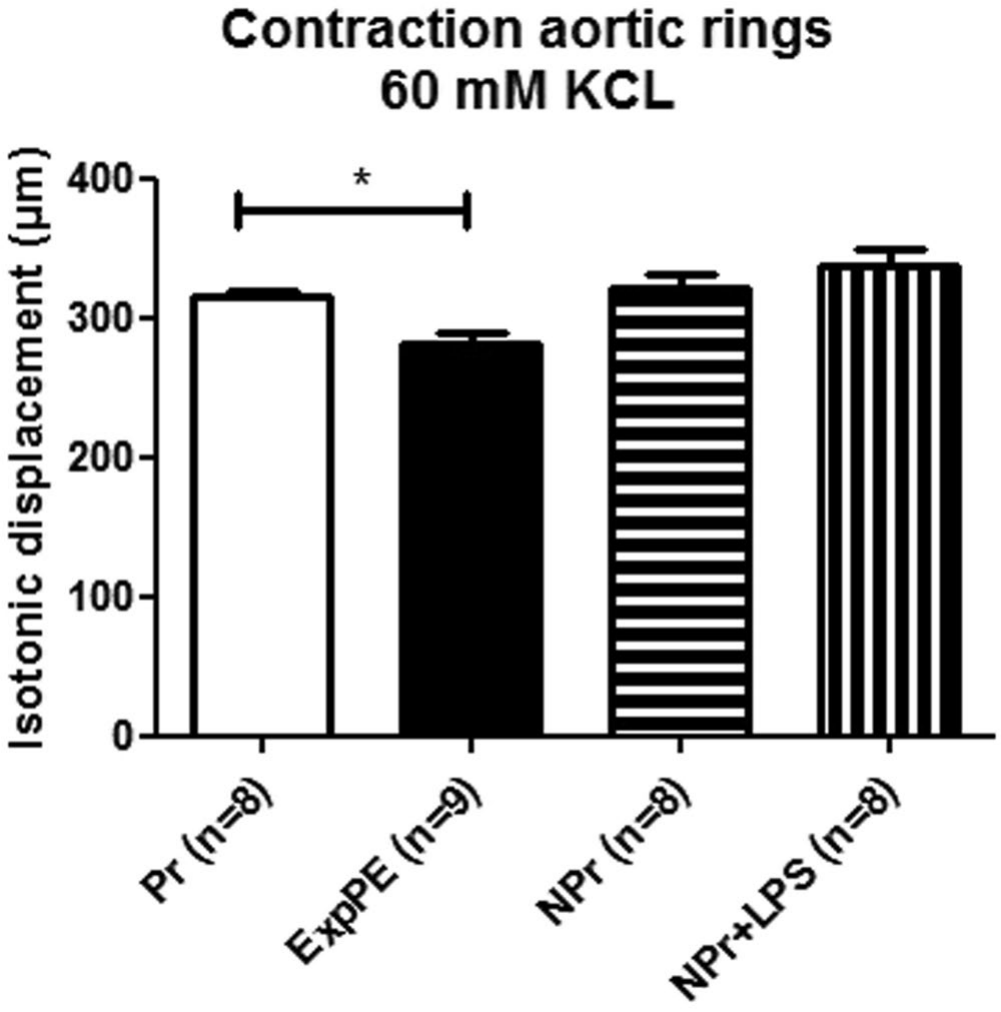
Contractility of aortic rings (isotonic displacement (microns)) after KCl (60 mM) incubation of 10 minutes. Aorta rings of Experimental Preeclamptic animals (ExpPE) had a significantly decreased response to KCl compared to healthy Pregnant (Pr) animal aorta rings. Non-Pregnant animals treated with LPS (NPr + LPS) did not differ in contractile aortic response after KCL incubation compared to Non-Pregnant animals without LPS treatment (NPr). Data are presented as mean ± SEM. * p < 0.05.

To examine functional changes of the aorta in response of potassium ions, aortic rings were incubated with KCl *ex vivo* and the contractility of the rings was measured. The contractile response of ExpPE aortic rings was significantly decreased compared to Pr rats (Fig. 5). Non-pregnant animals treated with LPS did not differ in contractile aortic response after KCL incubation compared to non-pregnant animals without LPS treatment.

## Discussion

**Figure 6:**
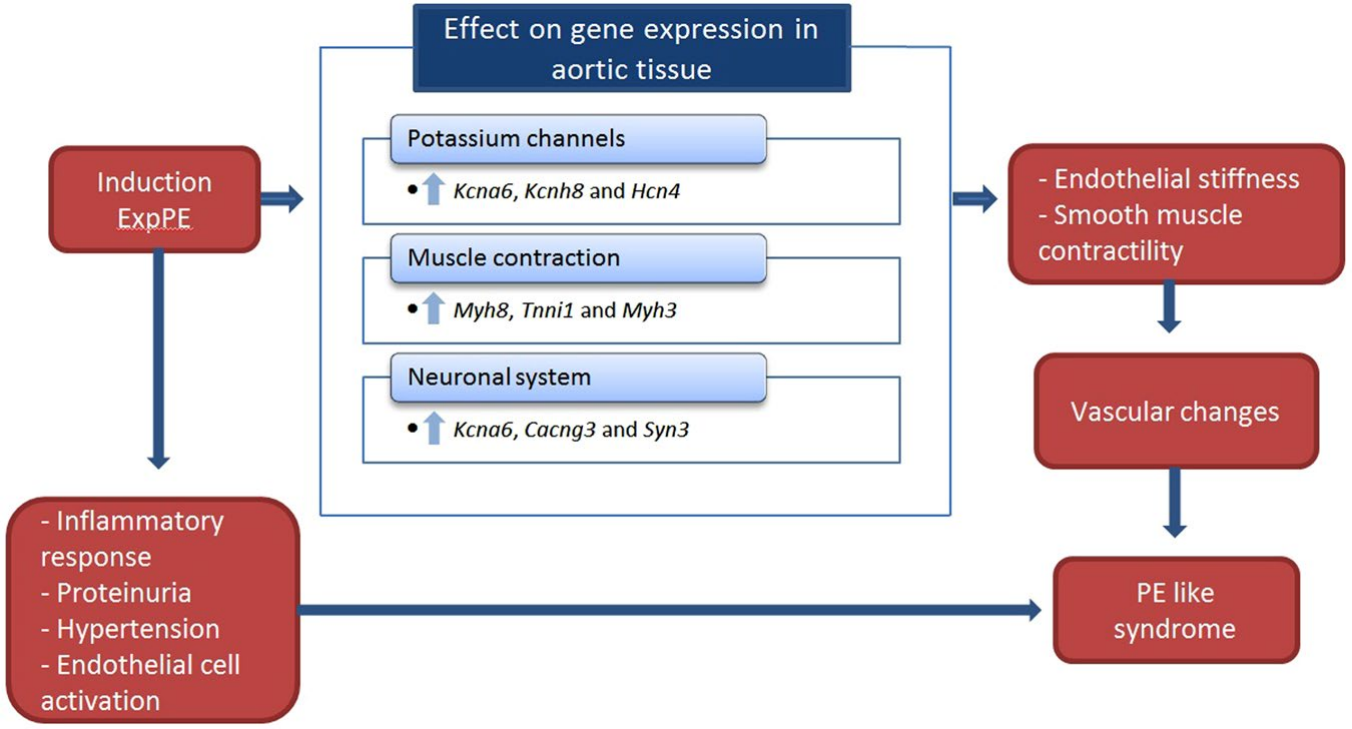
Schematic overview of the main findings and the hypothesis of the role of the findings. During pregnancy the experimental preeclamptic syndrome is induced, resulting in the main features of preeclampsia. Gene expression in the aorta is changed compared to healthy pregnant control animals which can lead to vascular changes in the animals and contribute to the preeclamptic syndrome. The three most contributing genes to the positively enriched gene sets are shown.

The aim of this study was to evaluate changes in the vascular transcriptome due to experimental preeclampsia in the rat. Therefore, we examined gene expression patterns in aortic tissue in non-pregnant, healthy pregnant and experimental preeclamptic rats by microarray technology. Eleven upregulated genes in ExpPE vs. Pr were validated by RT qPCR, and also evaluated in an additional control group of non-pregnant animals infused with LPS. This study showed that the gene sets “potassium channels”, “striated muscle contraction” and “neuronal system” were positively enriched in aortic tissue from preeclamptic rats vs. healthy pregnant rats (Fig. 6).

During pregnancy the maternal vascular system undergoes considerable adaptations, which are of importance for maternal health and fetal growth and development^20,21^. In the present study, we found pregnancy-induced adaptations in gene expression patterns in aortic tissue by comparing the Pr group with the NPr controls. We found that over 600 genes were differentially expressed in the aorta between the Pr and NPr group. The most notable changes were seen in genes associated with the immune system, such as the twelve-fold upregulation of *Cxcl13*, which encodes for a B-cell-attracting chemokine. *Mmp3*, which encodes for matrix metallopeptidase 3, was also upregulated in aortic tissue during Pr compared to NPr, which was also shown by Kelly *et al*.^22^. The *Mmp* family plays a role in vascular remodelling and angiogenesis by the degradation of extracellular matrix^23,24^.

GSEA was done to find functional changes in gene expression between the groups, with the use of predefined sets of genes. Each gene set encodes for one shared biological function, regulation or chromosomal location^25^. GSEA revealed that gene sets related to interferon (γ) signalling and cytokine signalling were highly positively enriched in the healthy pregnant group compared to NPr controls. Interferon γ is known to have a pro-inflammatory effect on the endothelium by upregulation of surface adhesion molecules and chemokines, such as CXCR3, CCR5, and CX3CR1 ligands^26^. Cytokine signalling in the endothelium may play an important role in the immune system and could be related to important changes in the immune system necessary for a healthy pregnancy^27,28^.

Experimental preeclampsia in rats induced with low-dose LPS during pregnancy is one of the main models to study the effects of preeclampsia on both the mother and the offspring^15,18,29–33^. We choose to use the aorta, since the aorta is easily accessible and often used for studies of vascular function in pregnancy, including our own studies^15,34–36^. The aorta, however, is a typical conductance vessel, rather than a resistance vessel associated with blood pressure regulation. Although the present study showed differential regulation of various genes and gene sets between the three groups of rats, in future studies we will need to confirm the role of these gene sets in hypertension and vascular function in resistance vessels.

The comparison of ExpPE with Pr revealed that over 500 genes were significantly (p < 0.05) differently expressed in the aorta with a relatively high fold change (>1.4 or <−1.4). The most upregulated genes in ExpPE compared to Pr were *Ttn* (Titin) and *Tnni1* (troponin I type 1 [skeletal, slow]), which are both important in muscle organization^37,38^. While the protein Titin is mostly known for its expression in skeletal muscle, it is also expressed in smooth muscle of the aorta^37^. Granzier *et al*.^39^ hypothesize that Titin could influence structural integrity and passive elasticity of smooth muscle tissues by linking dense bodies to thick filaments. Titin is also associated with heart failure with preserved ejection fraction by influencing the elasticity of myocardial muscle^40,41^. An increase of certain isoforms of Titin, which is also detected in a spontaneously hypertensive rat model^42^, correlates with increased passive stiffness of muscle tissue^39,43^. We are the first to hypothesize a role of increased Titin in hypertension in relation to preeclampsia. Multiple other genes associated with vascular function (*Nos1*, *Edn3* and *Ang2*) were also upregulated in ExpPE.

The most downregulated genes were *Nlrp1b* (NLR family, pyrin domain containing 1B) and *Ccl11* (chemokine [C-C motif] ligand 11). The proteins encoded by these genes both play a role in inflammation. *Nlrp1b* also plays a role in programmed cell death. Also potentially interesting is #10 in the list of downregulated genes, *Wnt16* (Wnt Family Member 16). The Wnt family is implicated in various developmental processes^44^. Downregulation of *Wnt16* is associated with vascular calcification^45^, which plays a role in vascular stiffness and is associated with hypertension^46^. The protein Wnt16 has been reported in circulatory vesicles of healthy pregnant women, while it was not detected in preeclamptic women^47^. If this gene is downregulated in preeclamptic patients, it may contribute to the development of artery calcification, which is observed in formerly preeclamptic women later in life^48^.

We performed a GSEA analysis in order to evaluate which gene sets are up- or downregulated in the aorta of preeclamptic rats. We showed that the most positively enriched gene sets were “potassium channels”, “striated muscle contraction” and “neuronal system”. The first 16 most highly contributing genes for the positive enrichment of the potassium channel gene set are significantly upregulated in ExpPE compared to Pr, 11 of these 16 genes are significantly upregulated in ExpPE compared to NPr controls. Since none of these genes are different between Pr and NPr animals, the effect of experimental preeclampsia on potassium channel gene expression is specific for experimental PE and not induced by pregnancy. Potassium channels play an important role in the vasculature by the establishment of the membrane potential^49^. The membrane potential determines the depolarization/repolarization state of cells, which affects the contractility of vascular smooth muscle cells^49,50^. Endothelial cells also express potassium channels^51^. The channels regulate the endothelial cell membrane potential and are, via Ca^2+^ signalling, involved in the production and release of endothelial derived vasoactive factors, such as nitric oxide, prostaglandins and EDHF^52^.

A role of potassium channels in aortic contraction in experimental preeclampsia may be in line with previous studies from our lab in the same model, in which we found an increased effect of EDHF and a decreased effect of prostaglandins in endothelial acetylcholine induced vasodilation in the aorta of preeclamptic rats vs. the aorta of healthy pregnant rats^15^. In this same study, we also found increased angII sensitivity in the aortas of preeclamptic rats. Since both EDHF and prostaglandins^53^, but also angII^54,55^ may affect vascular function via potassium channels, potassium channels may play a central role in the endothelial dysfunction in this model. This is also obvious from our *ex vivo* experiment in which aortic rings were treated with potassium chloride to induce contraction. We found decreased contraction in the aortic rings from preeclamptic rats, which may also suggests a different function of potassium channels in aortas of preeclamptic rats. Further studies are, however, needed to show the role of the increased expression of potassium channel genes in the decreased response to potassium in the aortas of rats with ExpPE, since potassium induced contraction in the *ex vivo* aortic contraction experiment may be due to membrane depolarization activating voltage operated calcium channels, inducing the influx of calcium into the cells and thereby contraction^56^. Our suggestion of a role for potassium channels in the hypertension in preeclampsia, seems to be in line with data from previous studies. It has been shown that during hypertension, ion channels are remodelled in the vasculature^57^, suggesting an important role of ion channels in the modulation of vascular tone. In line with the role of increased expression of potassium channels in hypertension, Cox *et al*.^58^ found found increased expression levels of some potassium channel genes in the hypertensive animals as compared with normotensive animals^58^. Other studies with hypertensive rat models also showed that the expression of potassium channel genes influence vascular dysfunction^59^ or hypertension^60^, though in these two studies a decrease or inhibition of a potassium channel gene induces this effect. Since upregulation of potassium channels is often associated with vasodilation^49^ we speculate that the increase in genes encoding for the gene set potassium channels could be an compensatory mechanism (which is insufficient in the present model), related to hypertension induced by other mechanisms, such as sympathetic activation^61^. The exact role of potassium channels in our model therefore needs further investigation.

Although our results strongly indicate a role of potassium channels in the pathogenesis of the present model, it remains to be established whether the changes in potassium channels also occur in preeclampsia. Watanapa *et al*.^62^ suggest such a role for potassium channels in preeclampsia, since they showed changes in potassium currents after incubation of endothelial cells with human preeclamptic plasma. It appeared that due to incubation with plasma, the inward K + currents were decreased compared to stimulation with plasma from healthy pregnancy, which may result in endothelial cell dysfunction and in a reduced production and release of vasodilators of endothelial cells^62^.

Next to the potassium channels gene set, the neuronal system gene set and striated muscle contraction gene set were also highly upregulated. Although in our model we did not study sympathetic activity, the upregulation of genes important in the neuronal system may suggest that neuronal genes affect vascular function in this model of experimental preeclampsia. The autonomic nervous system innervates the vascular wall and mediates vascular tone^63^: increased sympathetic activation, which is part of the autonomic nervous system, is strongly correlated with human hypertension^64^ as well as with hypertension during pregnancy^65^. Upregulated genes in the neuronal system could indicate an increased sympathetic activity resulting in vasoconstriction, contributing to the hypertension in our model. This data seem to be in line with the suggestion of Schobel *et al*. stating that during preeclampsia over-activity of the sympathetic system occurs^66^.

The gene set for striated muscle contraction was also upregulated. Signalling pathways in striated muscle contraction (including skeletal and cardiac muscle contraction) have many similarities with signalling pathways in smooth muscle contraction^67^. Furthermore, smooth muscle cells of the embryonic dorsal aorta, which progresses into the descending aorta, have a common clonal origin with skeletal muscle cells^68^. So, in analogy with skeletal muscle^38,69–71^, we speculate that this gene set also plays a role in smooth muscle contraction of the aorta and thus contributes to the hypertension in our model.

Although we did not include non-pregnant LPS rats in the array, we used RT qPCR to test the expression of the 11 most relevant upregulated genes in ExPE vs Pr rats. In line with our expectation that LPS would not affect gene expression in non-pregnant rats, nine out of eleven genes did indeed not respond to LPS in non-pregnant animals. Only the genes *Kcnq3* and *Cacng3*, increased upon LPS infusion in non-pregnant animals. Thus, the results suggest that most, but not all, genes differentially expressed in the preeclamptic animals are pregnancy specific and related to the preeclamptic state in the rat.

In conclusion, our data showed that experimental preeclampsia in rats resulted in changes in gene expression levels in the aorta compared to healthy pregnant rats. The data suggest that in the present model potassium channels and innervation as well as (striated) muscle contraction in the aorta may play a role in the pathophysiology. Whether similar changes take place in the vasculature in human preeclampsia remains to be established. We are currently preparing experiments in which cultured human endothelial and vascular smooth muscle cells are incubated with human preeclamptic and healthy pregnant plasma, followed by gene expression measurements. Our findings may contribute to a better understanding of the effects of the preeclamptic syndrome on the maternal vasculature. It may also help to explain the long-term effects of preeclampsia on the increased incidence for heart and vascular disease.

## Materials and Methods

### Animal model

Animal material was used from previously conducted experiments^15^. The use of animals was approved (application number: DEC-5516A) by the Ethical Committee for Animal Experimentation of the University of Groningen and animal experiments were performed in accordance with the National Institutes of Health Guide for the Care and Use of Laboratory Animals. Wistar outbred rats (Harlan Inc, Horst, the Netherlands) were housed in a 12-hour light-dark cycle with food and water ad libitum. A cannula was placed into the right jugular vein in animals at day 0 of pregnancy and also in age matched non-pregnant control animals while anesthetized with isoflurane/oxygen.

Animals were infused with either LPS (E-Coli, 0.55: B5, Whittaker MA Bioproducts, Walkerville, Md.) or saline 14 days after cannula placement. Experimental preeclamptic rats were infusede with LPS for 1 hour with 1 μg/kg bodyweight dissolved in 2 ml saline (n = 9). The Pr control animals received saline only (2 ml during 1 h; n = 8).

At day 20 of pregnancy, the animals were euthanized by decapitation and thoracic and abdominal aortas were isolated and cleaned from surrounding tissue. Non-pregnant female rats with saline (n = 8) or LPS (n = 8) infusion were euthanized on diestrus, and aortas were isolated and cleaned from surrounding tissue. The thoracic aortas were placed in cold oxygenated Krebs solution and prepared for contraction experiments. Abdominal aortas were stored at −80 °C until further use for microarray analysis.

### RNA isolation

Total RNA was isolated from whole abdominal aortas with TriReagent (Sigma-Aldrich, St. Louis, MO) following the manufacturer’s instructions from all groups of rats. An additional round of purification was performed with RNeasy Microkit columns (Qiagen, Venlo, the Netherlands). RNA quality was assessed using RNA 6000 nanochips on the Agilent 2100 bioanalyzer (Agilent Technologies, Amsterdam, the Netherlands), and all samples showed intact 18 S/28 S bands.

### Microarray

The microarray was performed with three animal groups: NPr (n = 4), Pr (n = 5) and ExpPE (n = 5). Total RNA (100 ng) was labelled using the Affymetrix WT plus reagent kit and hybridized to whole genome Genechip Rat Gene 1.1 ST arrays coding 19.357 genes (Affymetrix, Santa Clara, CA). Sample labelling, hybridization to chips and image scanning was performed according to the manufacturer’s instructions.

### Microarray data analysis

Microarray analysis was performed using MADMAX pipeline for statistical analysis of microarray data^72^. Quality control was performed and all arrays met our criteria. For further analysis a custom annotation was used based on reorganized oligonucleotide probes, which combines all individual probes for a gene^73^. Expression values were calculated using robust multichip average (RMA) method, which includes quantile normalisation^74^. Significant differences in expression were assessed using paired Intensity-Based Moderated T-statistic (IBMT^75^). All microarray data are MIAME compliant and have been submitted to the Gene Expression Omnibus (accession number GSE96610). Gene expression differences between the groups were considered significant with a p-value < 0.05 and a fold change <−1.4 or >1.4.

Gene Set Enrichment Analysis (GSEA) was performed comparing the three groups using MADMAX. In GSEA predefined sets of genes, which encode for one shared biological function, chromosomal location or regulation, are investigated and compared between the groups^25^. This way, functional changes in gene expression between the groups could be found.

The dataset generated and analysed during the current study is available at the Gene Expression Omnibus (accession number GSE96610).

#### Validation of the array

To verify microarray data, RNA was used from aorta of NPr (n = 4), Pr (n = 5) and ExpPE (n = 5) and RT qPCR was performed as described below on 11 genes. The 11 genes chosen were of most interest because they include: the top 2 upregulated genes in ExpPE vs. Pr which were also in the “striated muscle contraction” gene set. Two additional genes of the same gene set were also included, the top 5 mostly contributing genes to the enrichment of the gene set “potassium channels”, and the top 3 genes of the “neuronal system”.

#### Inclusion of the non-pregnant LPS treated animals

We did not include samples from non-pregnant rats treated with LPS on the array, since previous research showed no physiological differences due to low-dose LPS infusion in non-pregnant animals^15,16,18,30^. Instead, RT qPCR was used to measure gene expression in NPr + LPS rats (n = 5). We used the same 11 genes as for the validation of the array.

### RT qPCR

A total of 1 µg RNA was reverse transcribed using random nonamers (Sigma) and 1 µL (200 units) M-MLV RT (Invitrogen), according to the manufacturer’s instructions. cDNA was stored at −20 °C until further use.

RT qPCR was performed using 2 µL of 20x diluted cDNA, 2.875 µL sterile water, 0.125 µL (10 µM) forward and reverse primer mix, and 5 µL SYBR Green PCR Master Mix (Life Technologies) and run in triplicates on a StepOnePlus™ Real-Time PCR System machine (Applied Biosystems) using the following program: 10 min 95 °C, followed by 40 cycles: 15 sec. 95 °C and 1 min 60 °C. Primers (Invitrogen) were designed using Primer3 and BLAST (Table 5). The expression levels were calculated based on a calibration curve and data were normalized to those of *36b4*. Significance was determined on log transformed data which was standardized to 1.0 for Pr, using the Student’s t-test to compare ExpPE with Pr, and to compare NPr with NPr + LPS. P < 0.05 was considered significant. The data are presented as mean ± SEM. The correlation between microarray and RT qPCR gene expression values was determined by Pearson correlation.

**Table 5:** Primers for RT qPCR.

Gene name	Entrez ID	Forward primer (5′ - >3′)	Reverse primer (5′ - >3′)
*Ttn*	84015	AGTCAGAGCTACAGGCAACC	TCCTTCAATCCTGATCCTTGGG
*Tnni1*	29388	CTCATCTGCACAGGAACCAAC	TCAGGCTCTTCAGCATGAGTTTA
*Myh3*	24583	TTCGCTACGACAGATGCTGA	CACAAAGTGTGGGTGAGTGG
*Myh8*	252942	GAGGCTGAGGAACAATCCAAC	TGCGTTTACTCTGCACTGATTT
*Kcna6*	64358	CTTGCCTCTGAGGGCTGTG	ATCCAGAATCCCCCGTCTCA
*Kcnh8*	246325	ATCCACTACGTCACCACCTG	ATGTACGAGGGACACCACTG
*Kcnq3*	29682	CAAGTACAGGCGCATCCAAA	TAGCAAATGTTTCCAGCAGCA
*Kcnj3*	50599	CGAGCATGCGGTTATTTCCA	GTGTCTGCCGAGATTTGAGC
*Hcn4*	59266	CGTGAGGGCGGATACTTACT	GTTCTTCTTGCCTATGCGGT
*Cacng3*	140724	TGCTTAGAAGGAGCTTTCCGA	ACACAGAGTCCCCCGAAAAA
*Syn3*	29130	AGTTGTGAGAAATGGCACCAA	AGCTGAGAGAACACCCAAGG

### Contraction assay of aortic rings

Thoracic aorta tissue of Pr (n = 8), NPr (n = 8), NPr + LPS (n = 8) and ExpPE (n = 9) was first cleaned of surrounding tissue and then cut into 2 mm rings, which were kept in Krebs solution (26) (37 °C and aerated 95% O_2_, 5% CO_2_). Isotonic contraction experiments were conducted with thoracic aorta rings according to the procedure described by Buikema *et al*.^15,76^. In brief: the aortic rings were equilibrated for 30 minutes. Thereafter, the rings were stimulated with potassium chloride (KCl) (60 mM) for 10 minutes and the contraction was evaluated by measuring isotonic displacements (microns). The data were analyzed using GraphPad Prism version 5.0 on a standard computer and presented as mean ± SEM. Significance was determined with a One-way ANOVA followed by a Student’s t-test.

## Electronic supplementary material

Supplementary Figure S1.

Supplementary Table S1

Supplementary Table S2

Supplementary Table S3
