# Supplementary material for: Experimental preeclampsia in rats affects vascular gene expression patterns
Source: Sci Rep. 2017 Nov 1;7:14807. doi: 10.1038/s41598-017-14926-4 (PMC5665945; doi:10.1038/s41598-017-14926-4)
Supplement: Supplementary file 1 — Supplementary Figure S1. [file 41598_2017_14926_MOESM1_ESM.doc]

Manuscript: **Experimental preeclampsia in rats affects vascular gene expression patterns**

Simone V. Lip, Anne Marijn van der Graaf, Marjon J. Wiegman, Sicco A. Scherjon, Mark V. Boekschoten, Torsten Plösch, Marijke M. Faas

**Supplementary Figure 1.** Validation of the microarray gene expression values. Real-time quantitative PCR (RT qPCR) was performed to validate the microarray data. Gene expression levels of 11 genes in total were evaluated and correlation between microarray and RT qPCR gene expression values were determined by Pearson correlation.
